# Predictors of dietary supplement use among female health workers in Tehran

**DOI:** 10.1186/2251-6581-12-26

**Published:** 2013-06-08

**Authors:** Fereshteh Baygi, Gity Sotoudeh, Mostafa Qorbani, Haleh Sadrzadeh-Yeganeh, Abbass Rahimi, Fariba Koohdani, Hamid Asayesh

**Affiliations:** 1 Endocrinology and Metabolism Research Center, Tehran University of Medical Sciences, Shariati Hospital, Tehran, Iran; 2 Department of Community Nutrition, School of Nutritional Sciences and Dietetics, Tehran University of Medical Sciences, Porsina streets, Ghods street, Tehran, Iran; 3 Department of Public Health, Alborz University of Medical Sciences, Karaj, Iran; 4 Department of Epidemiology, Iran University of Medical Sciences, Tehran, Iran; 5 Department of Epidemiology and Biostatistics, School of Public Health, Tehran University of Medical Sciences, Tehran, Iran; 6 Department of Cellular, Molecular Nutrition, School of Nutritional Sciences and Dietetics, Tehran University of Medical Sciences, Tehran, Iran; 7 Qom University of Medical Sciences, Qom, Iran

**Keywords:** Dietary supplement, Lifestyle, Health worker, Women, Body mass index

## Abstract

**Background:**

Despite the lack of evidence on the necessity of dietary supplements to meet nutrients requirements, the majority of people use them all over the world. This study aimed to investigate factors associated with supplement use in women who work in health centers in the city of Tehran.

**Methods:**

Five hundred sixty three female health workers participated in a cross-sectional study carried out in 2010 in health centers of Tehran. Weight, height, and waist circumference were measured and body mass index was calculated. Data on demographic characteristics, lifestyle, and dietary supplement use were collected by interviewing. The analysis was conducted using univariate and multivariate logistic regression (MLR) in SPSS version 16.

**Results:**

The prevalence of dietary supplement use was 53.8%. In univariate logistic regression model, age, education, husband’s education, duration of employment, and tendency for changing weight at the time of the study were statistically significant predictors (P < 0.05). After MLR analysis, education (OR: 1.11, 95% CI: 1.05-1.17) and duration of employment (OR: 1.04, 95% CI: 1.02-1.06) remained significant in the model. Women with higher education and longer duration of employment had more tendency to use nutrient supplements.

**Conclusion:**

Our findings showed that education and duration of employment were the most important predictors for taking dietary supplements in this population.

## Background

Despite the lack of evidence on the necessity of dietary supplements to meet nutrient requirements, the majority of people use them all over the world [1, 2]. Recent studies showed that estimated prevalence of dietary supplement use is higher than those derived from previous surveys [1–3]. While many dietary supplements are safe at certain periods for most people to use [4], there is growing evidence that some type of supplements can cause serious adverse effects or have no beneficial effects [5–7]. Nowadays, numerous types of supplements have been available in Iran and it seems that their usage is more popular than previous years. Health workers have an important role in educating people who attend the health centers and their nutritional and health behaviors may influence the attendants. Since there is no documentary on the frequency of use and the predictors of supplement intake among health workers, this study was performed to assess some demographic characteristics and life style factors associated with dietary supplement use in women who work in health centers of Tehran.

## Material and methods

This study was approved by the ethics committee of Tehran University of Medical Sciences (TUMS). Five hundred sixty three volunteered female health workers participated in this cross-sectional study conducted in 2010 in Tehran. All health centers in Tehran are affiliated to two main medical universities (Tehran and Shahid Beheshti). A single stage stratified cluster sampling method was used to select participants. For sampling, the list of all health centers (n=325) and the frequency of their employees were prepared, as the sampling frame. These centers were stratified by the two universities. Simple random sampling was employed with probability proportional to the size of each stratum. Forty three centers affiliated to Tehran University and 73 centers affiliated to Shahid Beheshti University were selected. Each center was considered as a cluster and all women who were working at each selected center were invited to participate in the study. Subjects reported information about demographic data and lifestyle. In addition, they were asked about their use of dietary supplement in the preceding month of the study and the reasons for taking the supplements. The individual’s perception of health status was determined by self-rated health status questionnaire asking this question “ how would you rate your overall health status: excellent, good, average, poor, or very poor?” The participants’ responses were then categorized in three levels of excellent / good, average, poor / very poor [8]. Physical activity was measured very briefly by asking a question about the level of physical activity of subjects in their own view.

Height was measured by a stadiometer (Seca, Germany) in a standing position with bare feet (precision 0.5 cm), and body weight was determined with subjects wearing light clothes and no shoes or socks, using an electronic balance. Body mass index was calculated as weight (kg)/ squared height (m^2^). Waist circumference was measured at the level of the midway between the lowest rib and the iliac crest.

### Statistical analysis

Data were analyzed using the SPSS 16 for Windows (SPSS Chicago, IL). The crude odds ratio (OR) were computed by using logistic regression test to establish the degree of association between examined factors and dietary supplement use. Multiple logistic regression (MLR) model was fitted to data to adjust for confounders. All variables that had P < 0.05 in the univariate analysis were fitted into the stepwise multivariate model. Results of the MLR are presented by OR and 95% confidence interval. P-values less than 0.05 were considered as statistically significant.

## Results

Mean (±SD) of subjects’ age and years of education were 36.92 ± 9.05 and 14.98 ± 3.25 years, respectively. Prevalence of dietary supplement use in subjects was 53.8%. The most used supplements in participants were Iron, Calcium-Vitamin D, Folic acid, Muitivitamin, Zinc, Omega3, Vitamin E, Multivitamin & mineral, and Thiamin (Table 1). The most important reasons for taking Iron, Folic acid, Calcium-Vitamin D, Omega3, and multivitamin-mineral supplements were as follows: disease treatment and health/nutritional status promotion. Zinc and Vitamin E supplements were mainly used because of their impact on hair and skin health, respectively. The estimated crude ORs for age (OR: 1.025, 95% CI: 1.006-1.044), education (OR: 1.086, 95% CI: 1.029-1.145), husband’s education (OR: 1.109, 95% CI: 1.047-1.176), and duration of employment (OR: 1.032, 95% CI: 1.01-1.05) were statistically significant (Table 2). Association between qualitative variables and dietary supplement use showed in Table 3. The estimated crude OR for trying to lose weight in the preceding year was statistically significant (OR: 1.43, 95% CI: 1.017-2.023). After MLR analysis, education (OR: 1.11, 95% CI: 1.05-1.17) and duration of employment (OR: 1.04, 95% CI: 1.02-1.06) remained significant in the model.

**Table 1:** **Frequency of supplement usage and prescription by physician in studied subjects**

Dietary supplement	Frequency of usage N (%)	Prescribed by physician N (%)
**Iron**	91 (16.2)	52 (57.1)
**Calcium-Vitamin D**	87 (15.5)	61 (70.1)
**Folic acid**	74 (13.1)	43 (58.1)
**Multivitamin-mineral**	55 (9.8)	29 (52.7)
**Zinc**	51 (9.1)	31 (62.0)
**Omega3**	42 (7.5)	26 (61.9)
**Vitamin E**	37 (6.6)	25 (69.4)
**Multivitamin**	28 (5.0)	40 (50.0)
**Thiamin**	26 (4.6)	22 (84.6)

**Table 2:** **Association between quantitative variables and dietary supplement use in univariate logistic regression model**

Variable	Supplement use		OR	95% CI
	YES	NO		
**Age (year)**	37.8 ± 8.8	35.8 ± 9.1	1.025*	1.006-1.044
**Education (year)**	15.37 ± 3.03	14.52 ± 3.4	1.086*	1.029-1.145
**Husband’s education (year)**	15.74 ± 3.28	14.4 ± 4.002	1.109*	1.047-1.176
**Duration of employment (year)**	12.6 ± 8.4	10.3 ± 8.5	1.032*	1.01-1.05
**Meal frequency/d**	2.8 ± 0.47	2.7 ± 0.49	1.192	0.844-1.84
**Snack frequency/d**	2.12 ± 1.066	1.95 ± 1.068	1.163	0.992-1.36
**BMI (kg/m** ^**2**^ **)**	25.71 ± 4.43	25.53 ± 4.46	1.015	0.967-1.065
**Waist (cm)**	83.96 ± 10.37	82.97 ± 10.2	1.009	0.993-1.026
**Gravity (no.)**	1.84 ± 0.91	1.89 ± 0.854	0.975	0.815-1.167
**Parity (no.)**	2.29 ± 1.22	2.32 ± 1.17	0.946	0.739-1.21

* p < 0.05.

**Table 3:** **Association between qualitative variables and dietary supplement use in univariate logistic regression model**

Variable		Supplement use		OR	95% CI
		YES	NO		
		N (%)	N (%)		
Marital status	Single	74(50.0)	74(50.0)	-----	
	Married	214(55.3)	173(44.7)	1.23	0.85-1.80
	Divorced/ widowed	15(53.6)	13(46.4)	1.15	0.51-2.59
Physical activity					
	No	63(49.6)	64(50.4)	-----	
	1-2 times per week	42(60.9)	27(39.1)	1.58	0.871-2.86
	≥3 times per week	198(54.0)	169(46.0)	1.19	0.795-178
Tendency for weight change at the study time					
	Keep weight	64(49.6)	65(50.4)	-----	
	Lose weight	221(55.8)	175(44.2)	1.283	0.861-1.91
	Gain weight	18(48.6)	19(51.4)	0.962	0.463-1.99
Try for weight change in last year					
	No task	114(49.1)	118(50.9)	-----	
	Lose weight	76(58.1)	127(49.1)	1.43*	1.017-2.023
	Gain weight	13(46.4)	15(53.6)	0.897	0.409-1.96
Eating breakfast	Always	270(55.2)	219(44.8)	-----	
	Occasionally	16(45.7)	19(54.3)	0.68	0.34-1.36
	Never	17(43.6)	22(56.4	0.62	0.32-1.21
Health status now	Excellent/good	154(52.6)	139(47.4)	-----	0.796-1.59
	Moderate	125(55.6)	100(44.4)	1.12	0.550-1.93
	Bad/very bad	24(53.3)	21(46.7)	1.032	
Health status in past	Excellent/good	141(53.4)	123(46.6)	-----	
	Moderate	121(51.9)	112(48.1)	0.942	0.662-1.34
	Bad/very bad	41(62.1)	25(37.9)	1.43	0.823-2.48

* p < 0.05.

## Discussion

In the current study 53.8% of participants used dietary supplements. This rate is lower than the rate among women in California-USA [3] and much more than the rate among adults in Alberta [9], Korea [10] and Jordan [11]. In Norway 58% of adult women used dietary supplements whereas in Sweden 43% of women reported taking all kinds of supplements [12].

In older subjects who were employed for longer times, the prevalence of supplement use was higher. In MLR, both age and duration of employment were fitted into the model, and because of the co-linearity between these two variables, only employment duration, which was associated with age, remained significant. In Norway, Sweden and Denmark, similar to our study, the supplement use increased with age [12]. In a survey of British women in 33–72 years old age group, 61% of the participants took dietary supplements [13]. Foote and her colleague [14] showed that older individuals were more likely to use supplements. The findings of Frank et al. [15] are also similar to the results found in the present survey. It seems that supplement use may have already been an established behavior associated with increasing age. Iron and folate intake is more common among middle aged women susceptible to anemia while Calcium/vitamin D is taken more by older adults which are at higher risk for osteoporosis. This relationship was not assessed in present study.

In the present study, subjects with higher education were more likely to use dietary supplements; however, there was no relationship between husband’s education and subjects’ supplement use in multiple logistic regression. Gordon and her colleagues found that supplement use was significantly higher among women who had higher levels of education so that education beyond high school was the strongest predictor of using supplements [3]. Educational status and supplement use were assessed in a survey carried out in Denmark in 2001. The use of supplements increased with increasing length of education in similarity with Sweden and Norway [12]. This relationship might be explained by the increased concern and awareness of a healthy lifestyle among the classes with higher education, which is shown by choosing to take dietary supplements.

There is no relationship between physical activity and dietary supplement use. Our finding is in contrast with other studies that found higher use of dietary supplement among individuals who were more physically active [11, 16, 17]. In this study, physical activity was measured very briefly, which may not reflect actual physical activity level of the subjects. Based on our finding more investigation on physical activity and supplement usage is needed.

No association was observed between health status and supplement use; however, in other studies, subjects with good health status use more supplements [18, 19]. Gordon and her colleagues showed that some health conditions (arthritis, depression) were significantly associated with higher likelihood of using some kinds of supplements, while another (diabetes) was associated with lower likelihood of use [3]. We think the controversy between our results may be related to this concept that we did not ask the presence of any chronic health problem among subjects in detail and we know it may have affected the results.

We did not find any association between BMI and supplement use. But in some studies obesity was inversely associated with supplement use [14, 20, 21]. Investigators think this result might indicate a greater awareness of health and healthy lifestyle among normal weight and lean subjects than obese ones [14].

There was no association between marital status and supplement use but in a study of elderly Japanese men the results showed that married men were more likely to take vitamins than singles. The investigators of this study believed that family and friends can influence taking dietary supplements [22]. In a study performed by the University of Florida the results showed that those who are unmarried are more likely to use herbs than those who are married, but this difference is not seen in vitamin or mineral supplement use [23].

This study had some limitations which may have influenced the findings. First, Physical activity was measured very briefly and we think it may not reflect actual physical activity level of the subjects. Based on our findings more investigation on physical activity and supplement usage is needed. Second, we did not ask the presence of any chronic health problem from subjects in detail and it may have confounded our results. Despite this limitation, the current study provides some data on supplement use in a major group of women.

## Conclusion

Our findings revealed that women with higher education and employment duration were more likely to use dietary supplements. We suggest that priority be given to promote research on the safety and effectiveness of commonly used dietary supplements. Educational strategies to prevent the dietary supplement use without prescription by nutritionist or physicians are necessary. Public education for unsustainable use of dietary supplement should be targeted. Also development of guidelines for recommending dosages based on age, weight, and health history are strongly recommended.
